# Transabdominal Approach for Chylorrhea after Esophagectomy by Using Fluorescence Navigation with Indocyanine Green

**DOI:** 10.1155/2014/464017

**Published:** 2014-07-01

**Authors:** Takeshi Matsutani, Atsushi Hirakata, Tsutomu Nomura, Nobutoshi Hagiwara, Akihisa Matsuda, Hiroshi Yoshida, Eiji Uchida

**Affiliations:** ^1^Department of Gastrointestinal and Hepato-Biliary-Pancreatic Surgery, Nippon Medical School, 1-1-5 Sendagi, Bunkyo-ku, Tokyo 113-8603, Japan; ^2^Department of Surgery, Nippon Medical School Tama-Nagayama Hospital, 1-7-1 Nagayama, Tama, Tokyo 206-8512, Japan

## Abstract

A 70-year-old man who underwent two sessions of thoracoscopy-assisted ligation of the thoracic duct to treat refractory chylorrhea after radical esophagectomy for advanced esophageal cancer received conservative therapy. However, there was no improvement in chylorrhea. Then, transabdominal ligation of the lymphatic/thoracic duct at the level of the right crus of the diaphragm was performed using fluorescence navigation with indocyanine green (ICG). The procedure successfully reduced chylorrhea. This procedure provides a valid option for persistent chylothorax/chylous ascites accompanied by chylorrhea with no response to conservative treatment, transthoracic ligation, or both.

## 1. Introduction

Chylorrhea including chylothorax is uncommon after esophagectomy, with an incidence of 1% to 4% [1–3]. When conservative treatments fail to stop the leakage of chyle, surgical treatment is necessary to avoid increased morbidity and mortality. Current surgical options for ligation of the lymphatic/thoracic duct include the transthoracic approach [4–7] and the transabdominal approach [8–11]. However, the site of a chyle fistula is often difficult to detect intraoperatively because of inflammation and edema. Recently, intraoperative indocyanine green (ICG) fluorescence lymphography was introduced to exactly define the site of a fistula causing chylorrhea [4]. We report the usefulness of lymphatic/thoracic duct ligation by intraoperative ICG fluorescence navigation via a transabdominal approach.

## 2. Case Report

**Figure 1:**
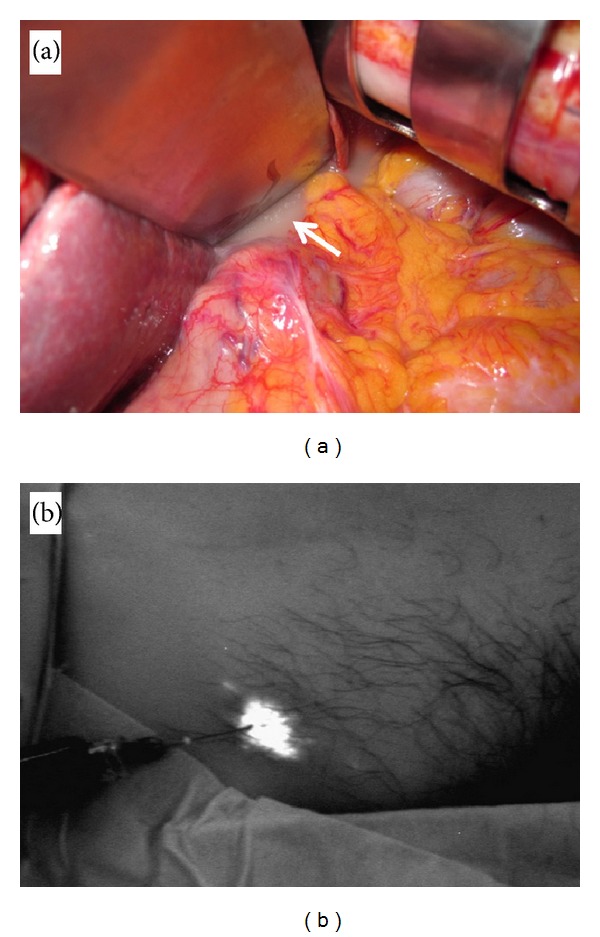
(a) The cisterna chili in the retroabdominal space below the diaphragm. (b) Indocyanine green is bilaterally injected subcutaneously into the inguinal region.

**Figure 2:**
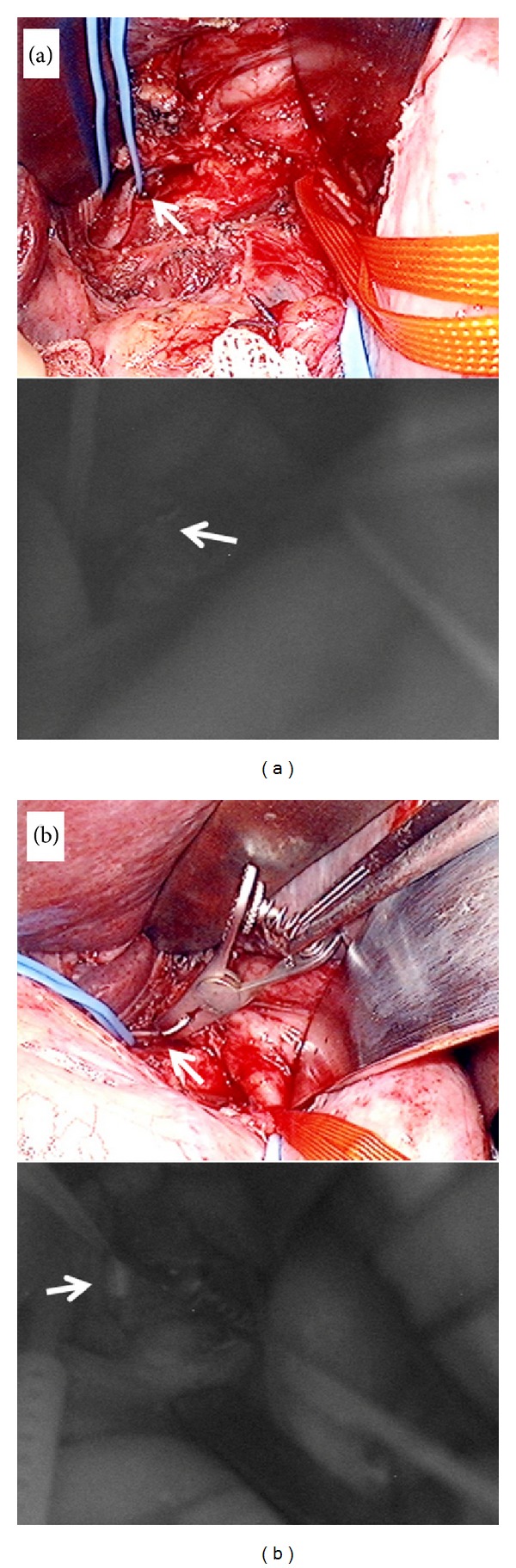
Intraoperative view and indocyanine green fluorescence lymphography. (a) The thoracic duct is seen on the right side of gastric tube (arrow). (b) A bulldog clamp is placed around the thoracic duct and the dilated thoracic duct is confirmed (arrow).

A 70-year-old man was referred to the hospital because of an advanced squamous cell carcinoma of the lower thoracic esophagus (T3N1M0, stage III). The patient received one course of neoadjuvant chemotherapy with 5-fluorouracil, docetaxel, and cisplatin. After a partial response was confirmed on repeated endoscopy and CT scan, the patient underwent a thoracoscopic subtotal esophagectomy with lymph node dissection in the prone position. Laparoscopy-assisted reconstruction was done using a gastric tube through the posterior mediastinal route with the patient in the supine position, combined with a jejunostomy. The thoracic duct from superior mediastinum to the diaphragm was resected with the esophagus, and the stump of the residual thoracic duct was clipped twice. Although the postoperative vital signs were stable, the chest-drain output continued to exceed 1500 mL/day on postoperative day (POD) 5. When elemental nutrition was started through the jejunostomy, the fluid from the chest drain turned milky white, confirming the diagnosis of chylorrhea. Conservative therapy with octreotide (intermittent subcutaneous injection 100 *μ*g × 3/day) and total parental nutrition for 5 days failed to reduce chylorrhea. We decided to perform repeated thoracoscopy-assisted ligation of the thoracic duct with the patient in the prone position on POD 10. However, reconstructed gastric tube occupied the space to search the thoracic duct in the right thoracic cavity. Then, the thoracic duct was not detected and ligated successfully. On POD 28, we performed an open laparotomy to ligate the thoracic duct at the level of the aortic hiatus after the administration of milk cream through the feeding jejunostomy tube. On the exploration of the upper abdomen, a milky effusion, chylous ascites, was found in the retroperitoneal space below the diaphragm (Figure 1(a)). The gastric tube was mobilized to the left side, but the exact site of the lymph fistula could not be identified. Approximately 10 min after a bilateral subcutaneous injection of 1.5 mL ICG (Diagnogreen 0.5%, Daiichi Sankyo Co., Tokyo, Japan) into the inguinal region (Figure 1(b)), the lymphatic duct was confirmed on fluorescence imaging performed with a near-infrared PDE camera system (Hamamatsu Photonics, Hamamatsu, Japan) at a 760 nm wavelength, filtering out light with a wavelength below 820 nm. The lymphatic duct, communicating with the thoracic duct, was visible at the level of right crus of the diaphragm (Figure 2(a)). A bulldog clamp was placed around the lymphatic duct, thereby confirming the dilated lymphatic duct as cisterna chili (Figure 2(b)). The root of thoracic duct was clipped successfully and easily at the right side of the esophageal hiatus. The chylorrhea stopped completely on POD 30. The postoperative course was uneventful. After the patient was able to ingest a solid meal, he was discharged on POD 35.

## 3. Discussion

Despite careful ligation of the thoracic duct following esophagectomy for esophageal cancer, the patients who undergo an extended lymphadenectomy for positive lymph node metastases have increased risk of chylorrhea by secondary injury to the main thoracic duct. A meta-analysis of 44 studies showed that chylorrhea developed in 2.1% of patients after transhiatal esophagectomy and 3.4% after transthoracic esophagectomy [12]. Systemic review and institutional analysis showed that postoperative chylorrhea occurred significantly more frequently among patients who had surgery after neoadjuvant chemotherapy than among patients undergoing surgery alone [13]. Loss of chyle leads to impaired wound healing due to loss of protein, lack of weight gain due to loss of fat, or immunodeficiency due to loss of lymphocytes. Early diagnosis and effective management are therefore necessary for good outcomes. However, the treatment of chylorrhea is controversial, and the choice between conservative therapy and surgery remains a matter of debate. Conservative therapy includes pleural drainage, parenteral nutritional support, and measures that reduce chyle flow, such as administration of medium-chain triglycerides and somatostatin analogs [2, 5, 14]. Although such conservative methods can avoid the need for surgery, they usually require several weeks for complete resolution of chyle flow [2, 5]. Thus, early operative intervention remains a good alternative for some physicians [6, 9]. In our patient, extended lymphadenectomy might have damaged the lymphatic system and caused persistent chylorrhea, despite careful ligation of the thoracic duct at initial operation.

Lymphangiography has traditionally been considered the gold standard for the evaluation of chylorrhea. This imaging technique is useful for preoperatively estimating the extent of lymphadenectomy. However, lymphangiography is a difficult procedure that requires cannulation of lymphatic channels, which can cause adverse effects such as local tissue necrosis, fat embolism to the lungs, hypersensitivity reactions, or worsening of lymphedema due to the contrast medium. To intraoperatively detect chyle leakage points, the administration of cream containing methylene blue dye via the oral or nasogastric route can facilitate leak identification [2, 6, 13]. A recent study reported that the precise site of chyle leakage was successfully detected on imaging by ICG fluorescence [4]. ICG particles are small and easily taken into lymph ducts. In addition, ICG binds to blood lipoproteins and shows diffuse fluorescence after excitation by near-infrared light. The photodynamic eye and fluorescence detector used in our patient were equipped with a light-emitting diode (760 nm) and CCD camera. Intraoperative ICG injection has no known adverse effects. Our findings confirmed that intraoperative ICG fluorescence lymphography can help surgeons detect sites of lymphatic duct injury. However, surgeons should know that the side effects by ICG administration as anaphylactic reaction, hypotension, tachycardia, dyspnea, and urticaria only occurred in individual cases; the risk of severe side effects rises in patients with chronic renal dysfunction.

The ligation of the thoracic duct through a second right-sided thoracotomy may be difficult not to detect the thoracic duct above the diaphragm after radical lymph node dissection. Moreover, it is likely that chyle was not leaked from one injury of the thoracic duct, from multiple lymphatic injuries in the upper abdominal cavity. A recent study reported that laparotomy with ligation of the thoracic duct at the level of the aortic hiatus is a simple and safe method for the management of postthoracotomy chyle leakage [9]. The study found that this technique is an effective treatment for postoperative chylorrhea; an abdominal approach has lower morbidity than a second transthoracic approach. Another advantage of a transabdominal approach is that surgeons can easily identify the lymphatic duct at the level of the hiatus because there are few anatomical variations of the thoracic duct, which is relatively consistent in the lower part of its course.

## 4. Conclusion

Intraoperative ICG fluorescence lymphography was useful for detecting the precise site of a lymphatic/thoracic duct injury after esophagectomy.
